# Music therapy in psychiatric units: evaluating its effectiveness

**DOI:** 10.1192/j.eurpsy.2023.1912

**Published:** 2023-07-19

**Authors:** M. Campillo, T. Vates, A. Pratdesava, M. Vallve, A. Casals, J. Ortega Vallve, R. Sanchez Gonzalez

**Affiliations:** 1 Psychiatry, Parc de Salut Mar, Santa Coloma Gramenet; 2 Music Therapy, Universitat Autonoma Barcelona, Barcelona, Spain

## Abstract

**Introduction:**

Research shows the benefits of music therapy for various mental health conditions, including depression, trauma, and schizophrenia. Music acts as a medium for processing emotions, trauma, and grief.

Playing instruments can encourage emotional expression, socialization and exploration of various therapeutic themes (i.e. conflict, communication, grief, etc.).

Group music therapy, measured by questionnaires and described in qualitative interviews, improved quality of life and self-esteem for people with severe mental illness (SMI).Group singing and song writing provide creative options for social connections. Music therapy should be considered as a component of holistic care for people with SMI. Jungup Lee, Thyer BA. May 2013Journal of Human Behaviour in the Social Environment 23(5):597-609

**Objectives:**

Music therapy sessions are held in our hospital for people admitted to short-term hospitalization units and to psychosocial rehabilitation units. The goal of the sessions is to create a connection space, promote people’s confidence in their own resources for their recovery, and evoke valuable experiences and memories. Sometimes musicians from the community have been present in the sessions, contributing to overcoming the stigma towards mental illness.

**Methods:**

We describe self-assessment of people admitted to psychiatric units after attending music therapy sessions. People from brief hospitalization unit filled out a survey, after each session, voluntarily, about their emotional state at the beginning of the session and after it. People from rehabilitation units, voluntarily filled the SRS V.3.0. 2002-Miller. Duncan & Johnson escale.The SRS was designed for use by clinicians to assess the therapeutic alliance during therapy (Duncan BL et al. The Session Rating Scale: Preliminary Psychometric Properties of a “Working” Alliance Measure JBT 3(1) 3-12 12/14/04 3:53 PM Page 3).

**Results:**

23 sessions took place for each unit. 39 patients from brief hospitalization, 22 women and 17 men, attended the sessions. 15 had a diagnosis of schizophrenia and related disorders, 13 were affective disorders, and 11 others diagnosis. All of them liked the participation either fully or partially. 76% men and 77% women felt better after, none of them reported to feel worse. 82% men and 86% women replied they would repeat the session.

Patients from rehabilitation units were 7 women and 10 men. 14 had a schizophrenia related disorder and 3 had bipolar disorder. All items on the scale were scored above 9 over 10, (*I felt heard, understood, and respected/ We worked on and talked about what I wanted to work on and talk about/ The therapist’s approach is a good fit for me*) with an overall score of 9,62 over 10 (*Overall, today’s session was right for me*).

**Conclusions:**

Music therapy sessions achieve benefits on an emotional level in any of the diagnoses, improving alliance with care teams, who value sessions as normalizing spaces, helping to overcome stigma.

**Disclosure of Interest:**

None Declared

